# SOX13 as a potential prognostic biomarker linked to immune infiltration and ferroptosis inhibits the proliferation, migration, and metastasis of thyroid cancer cells

**DOI:** 10.3389/fimmu.2024.1478395

**Published:** 2024-12-11

**Authors:** Yan-yan Ma, Wei-ye Zhou, Yue Qian, Ying-ying Mu, Wei Zhang

**Affiliations:** ^1^ Department of Rehabilitation Medicine, Beijing Jishuitan Hospital Guizhou Hospital, Guiyang, Guizhou, China; ^2^ Cell Biology Department, Wuxi School of Medicine, Jiangnan University, Wuxi, Jiangsu, China; ^3^ Department of Pathogen Biology, Guizhou Nursing Vocational College, Guiyang, Guizhou, China; ^4^ Department of Pathology, Zunyi Hospital of Traditional Chinese Medicine, Zunyi, Guizhou, China

**Keywords:** SOX13, biomarker, THCA, genetic alterations, tumor-immune infiltration, ferroptosis

## Abstract

**Background:**

SOX13 is a transcription factor belonging to the SOX family. SOX proteins are critical regulators of multiple cancer progression, and some are known to control carcinogenesis. Nevertheless, the functional and clinical significance of SOX13 in human thyroid cancer (THCA) remain largely unelucidated.

**Methods:**

Data on SOX13 expression were obtained through The Cancer Genome Atlas together with Gene Expression Omnibus. Co-expression, differential expression, and functional analyses of genes were investigated by databases. Associations between SOX13 levels, immune infiltration, ferroptosis, and immune checkpoint gene levels were analyzed. Genetic changes in SOX13 were investigated using CBioPortal. Associations between SOX13 levels and THCA clinicopathological features were analyzed and nomogram modeling for diagnostic and prognostic prediction. The influence of SOX13 on proliferation, migration, and metastasis was determined in KTC-1 and TPC-1 cell lines.

**Results:**

SOX13 was significantly lower in THCA tumors compared to controls. In addition, upregulated SOX13 gene mutation were evident in thyroid cancer. SOX13-associated genes exhibited differential expression in pathways associated with thyroid cancer development. Significant associations were found between SOX13 levels, immune infiltration, ferroptosis, and immune checkpoint genes in THCA tissue. SOX13 levels correlated with THCA stage, histologic grade, and primary neoplasm focus types, and independently predicted overall and progression-free intervals. SOX13 expression effectively distinguished between tumor and normal thyroid tissue. Spearman correlations highlighted a significant relationship between SOX13 and ferroptosis-associated genes. Overexpression of SOX13 enhances the inhibition of RSL3 (iron death activator) on the cell viability of TPC-1. Higher SOX13 levels in Thyroid cancer cells may lead to reduced proliferation, migration, and metastasis by regulating ferroptosis.

**Conclusion:**

Reduced SOX13 expression inversely impacts patient prognosis. In addition, SOX13 strongly regulates cancer immunity and Ferroptosis. Hence, SOX13 has great promise as a bioindicator for both thyroid cancer prognosis and immune cell invasion.

## 1 Introduction

Thyroid cancer is the leading endocrine cancer, and its incidence is increasing (1, 2). The disease arises from thyroid follicular epithelial cells, which constitute over 95% of thyroid malignancies. Types include papillary thyroid carcinoma (PTC), anaplastic thyroid carcinoma (ATC)/undifferentiated thyroid carcinoma (UTC), and follicular thyroid carcinoma (FTC) (3). Medullary thyroid carcinoma (MTC) derives from thyroid parafollicular cells and demonstrates a highly malignant transformation. According to 2021 data, the United States estimated 44,280 cases of thyroid cancer, comprising 12,150 males and 32,130 females (4–6). Despite 90% of patients achieving cure through standard treatment, approximately 10% experience local recurrence or distant metastasis, significantly impacting prognosis. Therefore, a comprehensive elucidation of thyroid cancer etiology and novel molecular biomarker or prognostic model identification are crucial for early detection and treatment strategies.

SOX13 is a member of the SOX family, spanning approximately 12 kb with 13 exons (7). It is expressed in humans and other animals such as mice, with the human SOX13 gene located on chromosome 1q32 (8). Research indicates that SOX13 plays crucial roles in preventing inflammation, promoting cartilage formation, neurogenesis (9, 10), limb development, and T-cell differentiation. Emerging evidences revealed that SOX13 is intricately linked to multiple cancers, namely, hepatocellular carcinoma, colorectal cancer, gastric cancer, and gliomas (11). Recent reports indicate that SOX9 plays a role in the invasiveness of thyroid cancer through the Wnt/β-catenin pathway (12). Both SOX9 and SOX13 belong to the same subfamily within the SOX gene family. However, whether SOX13 is involved in the regulation of thyroid cancer remains unexplored.

Herein, we assessed SOX13 levels and their correlation with patient prognosis in a THCA cohort. Additionally, we examined links between SOX13 content and immune cell invasion, ferroptosis, and immune status-indicating gene sets using several databases. Simultaneously, we assessed the impact of SOX13 overexpression on THCA cell proliferation, apoptosis, migration, and oncogenic signaling networks. The findings underscored the critical significance of the SOX13 gene in THCA prognosis and demonstrated its correlation with clinical outcomes in the THCA cohort, including tumor-invading immune cells.

## 2 Methodology

### 2.1 Datasets

Datasets, both clinical together with RNA-seq, from 512 THCA (TCGA-THCA) cases, together with information on 59 matched pairs of tumor/adjacent non-cancerous tissue were obtained through TCGA database. Datasets were collected within FPKM format and were subsequently converted to TPM using the following formula: $TPM=\frac{FPKM}{sum\;of\;FPKM\;values\;for\;all\;genes}\times 10^{6}$ . RNA-seq datasets within TPM format were also obtained via UCSC Xena from the GTEx database and analyzed using the DESeq2 package, which provides tools for quality control and normalization (13). The analyses followed the guidelines of the Declaration of Helsinki, 2013 revision. Information on SOX13 mRNA was also obtained from the GEO GSE33630 and GSE65144 datasets. The HPA databaseincludes immunohistochemistry (IHC) data from 44 different types of normal tissues and 17 major types of cancers (14).

### 2.2 TIMER2.0 analysis

The TIMER2.0 database allows the investigation of differential gene expression between tumor and control samples using TCGA data (15, 16). Thus, TIMER2.0 was used to determine SOX13 levels in various cancers.

### 2.3 Survival analysis

We analyzed survival data from 512 THCA patients obtained from The Cancer Genome Atlas (TCGA) database. Kaplan-Meier plots, together with log-rank assessments, probed survival, utilizing median level of SOX13 expression as a cutoff value. The links across clinical features and survival were assessed through univariate/multivariate Cox regression (17, 18) with major parameters (P< 0.05) from the univariate analysis employed within multivariate analysis. Forest maps were constructed using the R package “ggplot2”.

### 2.4 Mutation analysis of SOX13 in THCA

The frequencies of SOX13 mutations in THCA were assessed using cBioPortal and the specific types of mutation were also analyzed using the Catalogue of Somatic Mutations in Cancer (COSMIC) repository.

### 2.5 Functional enrichment analyses

Significant SOX13-related genes were identified by GO, KEGG and GSEA. GSEA utilized the gene set ‘h.all.v2022.1.Hs.symbols.gmt [Hallmarks]’ (19). Significance cut-off was adjusted to false discovery rate (FDR) < 0.25 and p.adjust < 0.05.

### 2.6 Immune invasion

Single-sample GSEA (ssGSEA) analyses were performed through R package GSVA package to examine THCA tumor infiltration of immune cells in relation to gene expression. Twenty-four immune cell types were investigated in terms of the gene expression profiles (20). Links across SOX13 levels and subsets of infiltrating cells were examined with Spearman and Wilcoxon rank-sum tests, and Pearson correlation coefficients evaluated associations between SOX13 and immune checkpoint genomic expression distribution. Associations between SOX13 levels and immune cells were examined by analysis of chemokines and their receptors using the”chemokine” module in TISIDB.

### 2.7 Relationships between SOX13 levels and ferroptosis-associated genes

The potential relationships between SOX13 and ferroptosis-associated gene levels were evaluated in TCGA-THCA datasets using DESeq2 package (version 1.30.1), which was also used to assess the proportions of ferroptosis-associated genes in high- or low-SOX13 samples. The “ggboxplot” package in R was used for visualization of the results.

### 2.8 Cell culture and cell experiment

KTC-1 and TPC-1 cell lines were employed to assess the SOX13-mediated regulation of THCA cells. The cells were acquired from Procell Life Science & Technology Co., Ltd (CL-0103, Wuhan, China) and were maintained in DMEM with 10% fetal bovine serum, 100 U/mL penicillin, and 100 U/mL streptomycin, at 37°C with 5% CO_2_ and saturated humidity in a constant temperature incubator.

### 2.9 Plasmid transfection

The SOX13-overexpression plasmid (OE-SOX13) and the negative control NC recombinant plasmid (NC-SOX13) (**Supplementary Table S1**) were constructed using Gima Gene (Shanghai, China). Plasmids and vectors were transfected into THCA cells with lipofectamine 6000 (Beyotime Biotechnology, Shanghai, China) or DNA transfection reagent (TF201201(TF20121201), Neofect (beijing) biotech Co,.Ltd, Beijing, China) as per kit directions.

### 2.10 Western blot

Following KTC-1 and TPC-1 cell lysis in RIPA buffer (P0013B, Beyotime Biotechnology) containing phenylmethanesulfonyl fluoride (PMSF, ST506) and phosphatase inhibitor (PPI, P1081), proteins were electrophoresed on SDS-PAGE prior to transfer to PVDF membranes (0.45 µm; Merck Millipore, Burlington, MA, USA). Membranes underwent a 1-hour blocking in 5% BSA. Subsequently, they were treated with primary antibodies rabbit anti-SOX13 (K007627P, Solarbo, Beijing, China; 1:1000), rabbit Anti-NFE2L2 (NRF2) Polyclonal Antibody (K106685P, Solarbo, Beijing, China; 1:1000), rabbit anti-TFRC (AF8136, Beyotime Biotechnology, Beijing, China; 1:1000), rabbit anti-SLC7A11 (AF7992, Beyotime Biotechnology, Beijing, China; 1:1000), rabbit anti-GPX4 (K003083P, Solarbo, Beijing, China; 1:1000), and rabbit anti-GAPDH (GB15002, Servicebio Biotech, Wuhan, China; 1:2000), followed by secondary antibodies. Protein quantification was done with Tanon-2500B gel imaging analysis system (Tanon, Shanghai, China), and signal intensity was measured with ImageJ (V1.6, NIH, Bethesda, MD, USA) using densitometry.

### 2.11 Wound healing assays

KTC-1 and TPC-1 cells underwent a 48-h transfection with SOX13-overexpression plasmid. Once confluent in 6-well plates, cells were PBS-rinsed to eliminate excess. Using a 200 µl pipette tip, scratches were generated on the monolayer and the cells were kept in serum-free medium with imaging at 0, 24, and 48 h and analysis with ImageJ 2.3.0.

### 2.12 Cell viability assays

KTC-1 and TPC-1 cells underwent transfection with SOX13-overexpression plasmid prior to a 48-h culture or RSL3(IR1120, Beyotime Biotechnology, Beijing, China) to a 24-h culture in 96-well plates (5x10^3^/well). Subsequently, 10 µL CCK-8 reagent (MCE, USA) was introduced to 100 µL medium in each well, and absorbances at 450 nm were read in a Bio-Tek microplate reader (Winooski, VT, USA) following 1 h of CCK-8 treatment.

### 2.13 Transwell invasion assay

The Transwell upper chamber contained 3 × 10^4 cells (100 µL) in serum-free DMEM, while the lower chamber included medium with 10% FBS (600 µL). The setup was incubated at 37°C for 30 h, prior to staining of invaded cells with crystal violet staining solution (C0121, Beyotime Biotechnology) and observed under a light microscope.

### 2.14 Ethics statement

Ethical approval was waived by the respective committee of Guizhou Nursing Vocational College as the tissue microarrays were commercially procured.

### 2.15 Statistical analyses

Bioinformatics data were assessed through R version 4.0.3. Datasets reflected means ± SEM, while differences were evaluated with t-tests or one-way analysis of variance (ANOVA) for separate specimens. P< 0.05 was deemed to confer statistical significance. Relationships between SOX13 levels and patient clinicopathological features were analyzed by χ2 assessments, logistic regression, Fisher’s exact, and Wilcoxon rank-sum assessments.

## 3 Results

### 3.1 SOX13 levels in THCA

**Figure 1:**
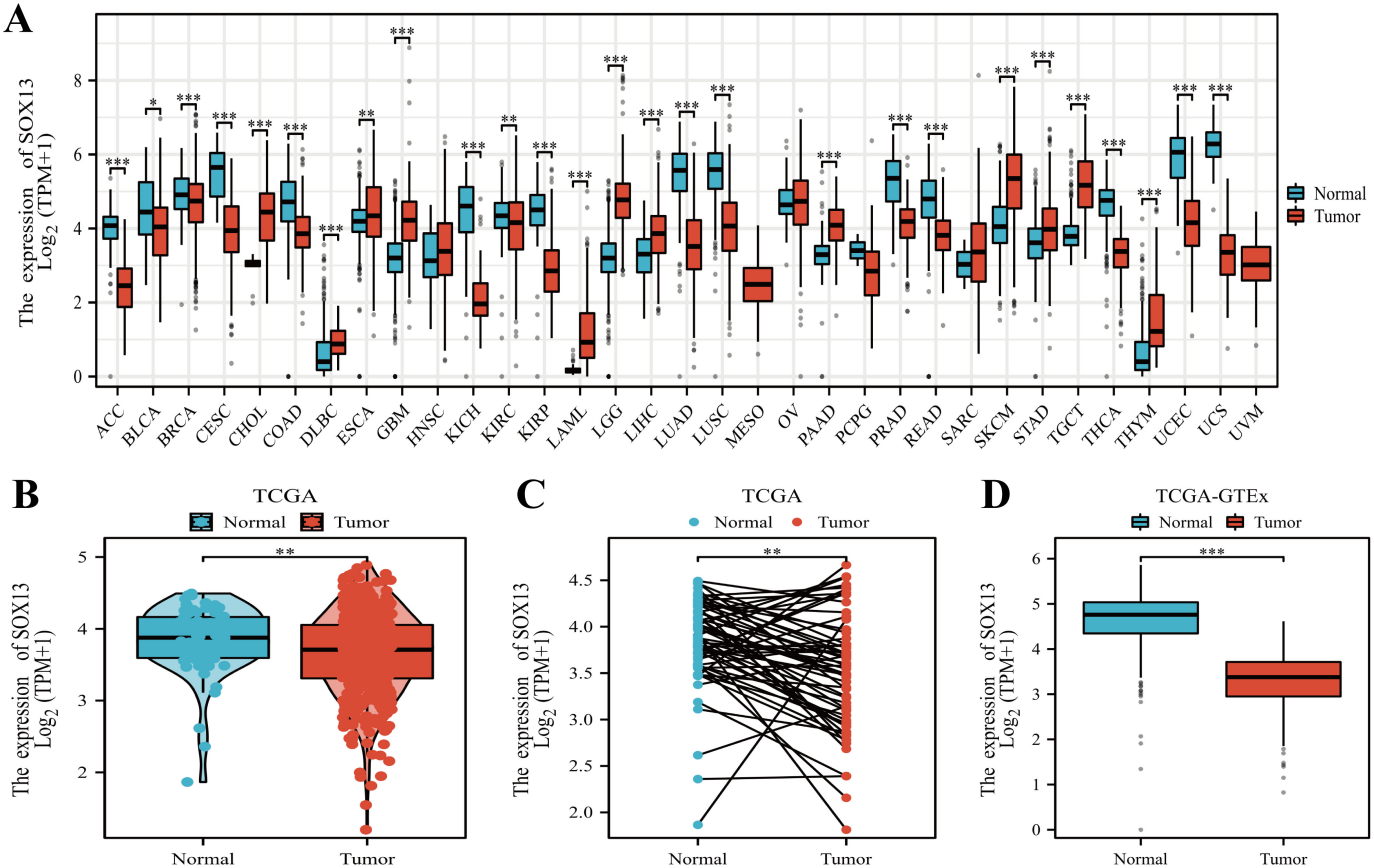
SOX13 expression profile across differing cancer models. **(A)** SOX13 levels within various tumors, determined through TIMER2.0^®^. **(B)** SOX13 levels across tumor/healthy specimens, from TCGA. **(C)** SOX13 levels in matched tumor and normal samples (TCGA). **(D)** Comparative analysis for SOX13 expression across GTEx and TCGA databases, evidenced by Wilcoxon rank-sum tests. *P< 0.05, **P< 0.01, ***P< 0.001.

We analyzed SOX13 expression across 38 cancer types using the TIMER2.0 database. SOX13 showed significant discreet expressions between tumor and normal groups across 20 types of malignancies, including THCA (P<0.001; **Figure 1A**). The relationships between SOX13 levels and clinical features were evaluated using TCGA data. This indicated that, in agreement with the above findings, SOX13 levels were markedly reduced in the 512 THCA tissues in comparison with the 59 controls (P< 0.01; **Figure 1B**). A further comparison of 59 paired THCA and normal tissues confirmed these findings, showing reduction of SOX13 in tumor tissues (P< 0.01; **Figure 1C**). SOX13 mRNA levels were then investigated in both TCGA and GTEx, showing marked reduction in 512 tumors compared with 338 control specimens (P< 0.001; **Figure 1D**).

**Figure 2:**
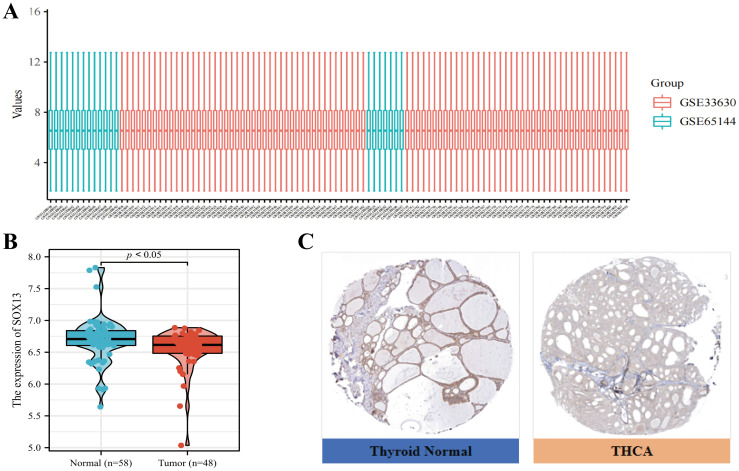
Levels of SOX13 in the GSE33630 and GSE65144 microarray datasets **(A)** Gene profile matrices were combined without inter-batch variations. **(B)** SOX13 profile scores in microarray datasets GSE33630 and GSE65144. **(C)** Immunohistochemical staining of SOX13 protein in THCA and normal thyroid tissues was performed with the HPA database.

Microarray dataset profiles GSE33630 and GSE65144 were downloaded from GEO DataSets. After normalization, probes were converted to gene symbols for series matrix files of each dataset and the gene expression data of these two datasets were merged (**Figure 2A**). The expressions of SOX13 in both datasets was tested and it was found that the expression level of SOX13 is significantly down-regulated in the 48 THCA tissues in comparison with the 58 controls (P < 0.05; **Figure 2B**). Furthermore, IHC staining data from HPA database indicated that medium levels of SOX13 expression were present in normal thyroid tissues, while low levels of expression were observed in THCA tissues (**Figure 2C**). Taken together, these results indicated that SOX13 was more highly expressed at the transcriptional and proteomic levels in normal thyroid tissues than in THCA tissues.

### 3.2 Analysis of prognostic factors and survival analysis of SOX13 in THCA

**Figure 3:**
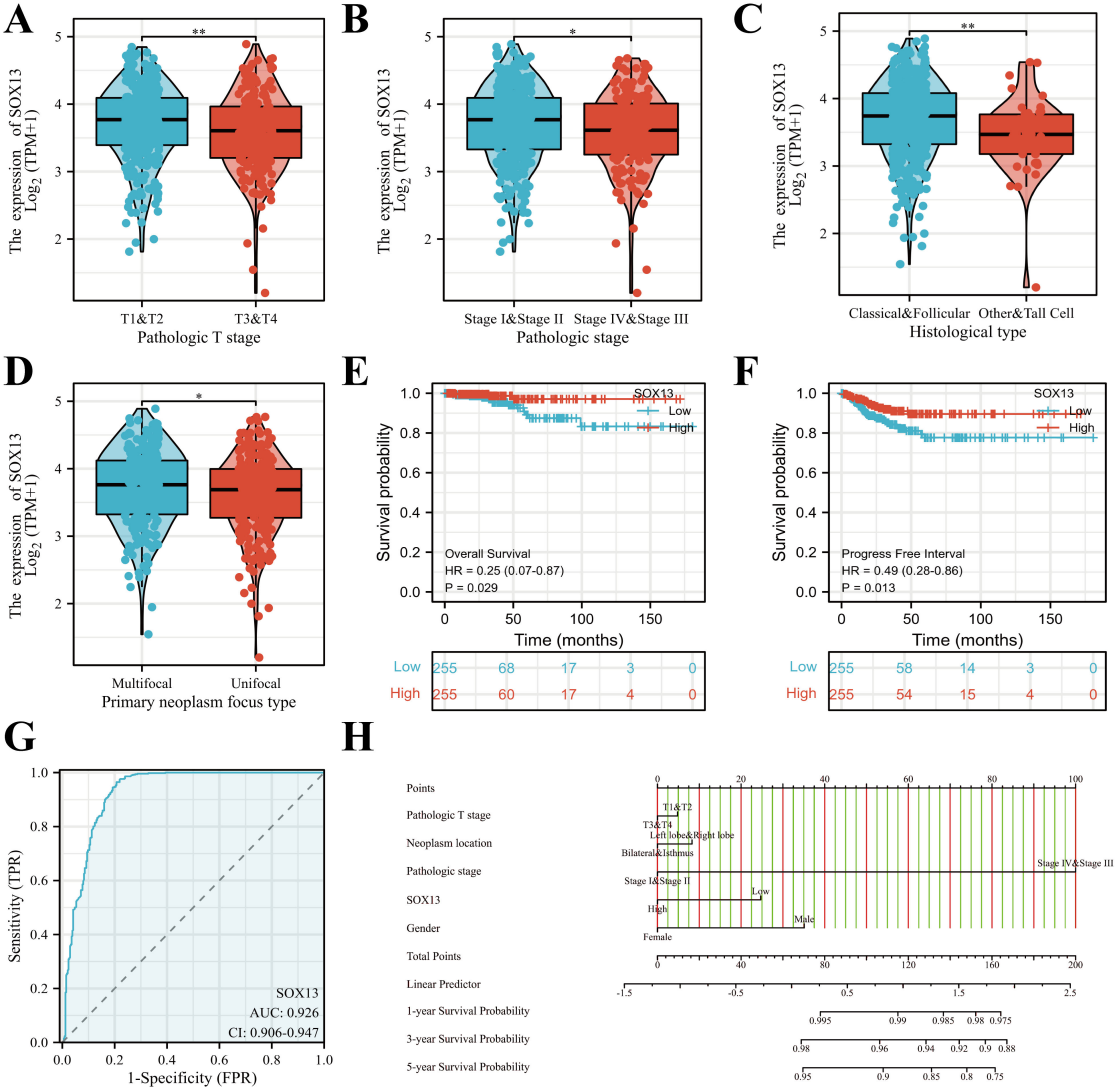
SOX13 mRNA levels in relation to clinical features and prognostic survival curve of THCA. **(A)** Relationship between SOX13 mRNA levels and T stage. **(B)** Links across SOX13 mRNA expression and pathologic stage. **(C)** Links across SOX13 mRNA levels and Histological type. **(D)** Links across SOX13 mRNA levels and Primary neoplasm focus type. **(E)** OS from the GEPIA database. **(F)** PFI from the GEPIA database. **(G)** The predictive performance of SOX13 (AUC). **(H)** Nomogram estimating the 1-, 3-, and 5-year OS of THCA patients. *P< 0.05, **P< 0.01.

Clinical information and SOX13 levels in 512 THCA patients from TCGA. Associations between these parameters were examined by univariate analysis, finding a significant association between reduced SOX13 levels and T and pathological stage, and histological and primary neoplasm focus types (all P< 0.05; **Figures 3A–D**, **Table 1**). The dataset outcomes suggested THCA cases with down-regulated SOX13 had raised odds for experiencing advanced disease in comparison with those with heighter SOX13 levels.

**Table 1:** Links across SOX13 levels and THCA clinicopathological characteristics, shown through logistic regression assessment.

Characteristics	Total (N)	OR (95% CI)	P value
Pathologic T stage (T3&T4 vs. T1&T2)	510	0.628 (0.439 - 0.900)	0.011
Pathologic N stage (N1 vs. N0)	462	0.671 (0.465 - 0.968)	0.033
Pathologic M stage (M1 vs. M0)	295	0.676 (0.178 - 2.570)	0.566
Pathologic stage (Stage III & Stage IV vs. Stage I & Stage II)	510	0.630 (0.435 - 0.914)	0.015
Neoplasm location (Left lobe & Right lobe vs. Bilateral & Isthmus)	506	0.650 (0.424 - 0.996)	0.048
Gender (Male vs. Female)	512	1.580 (1.066 - 2.342)	0.023
Histological type (Other & Tall Cell vs. Classical & Follicular)	512	0.374 (0.192 - 0.732)	0.004
Primary neoplasm focus type (Unifocal vs. Multifocal)	502	0.853 (0.600 - 1.212)	0.374
Thyroid gland disorder history (Normal & Other, specify vs. Lymphocytic Thyroiditis & Nodular Hyperplasia)	454	1.237 (0.832 - 1.841)	0.294

Kaplan–Meier survival plots demonstrated that cases having reduced SOX13 had significantly reduced OS (HR=0.25, P< 0.029) (**Figure 3E**), together with progression-free intervals (PFI) (HR=0.49, P< 0.013) in comparison to cases having upregulated SOX13 (**Figure 3F**). The ROC curve analysis showed that SOX13 had a satisfactory diagnostic value and the AUC of SOX13 were 0.926 in THCA (**Figure 3G**). A nomogram was plotted to determine the eligibility of SOX13 in predicting survival duration of THCA patients (**Figure 3H**).

### 3.3 Mutations of SOX13 in THCA

**Figure 4:**
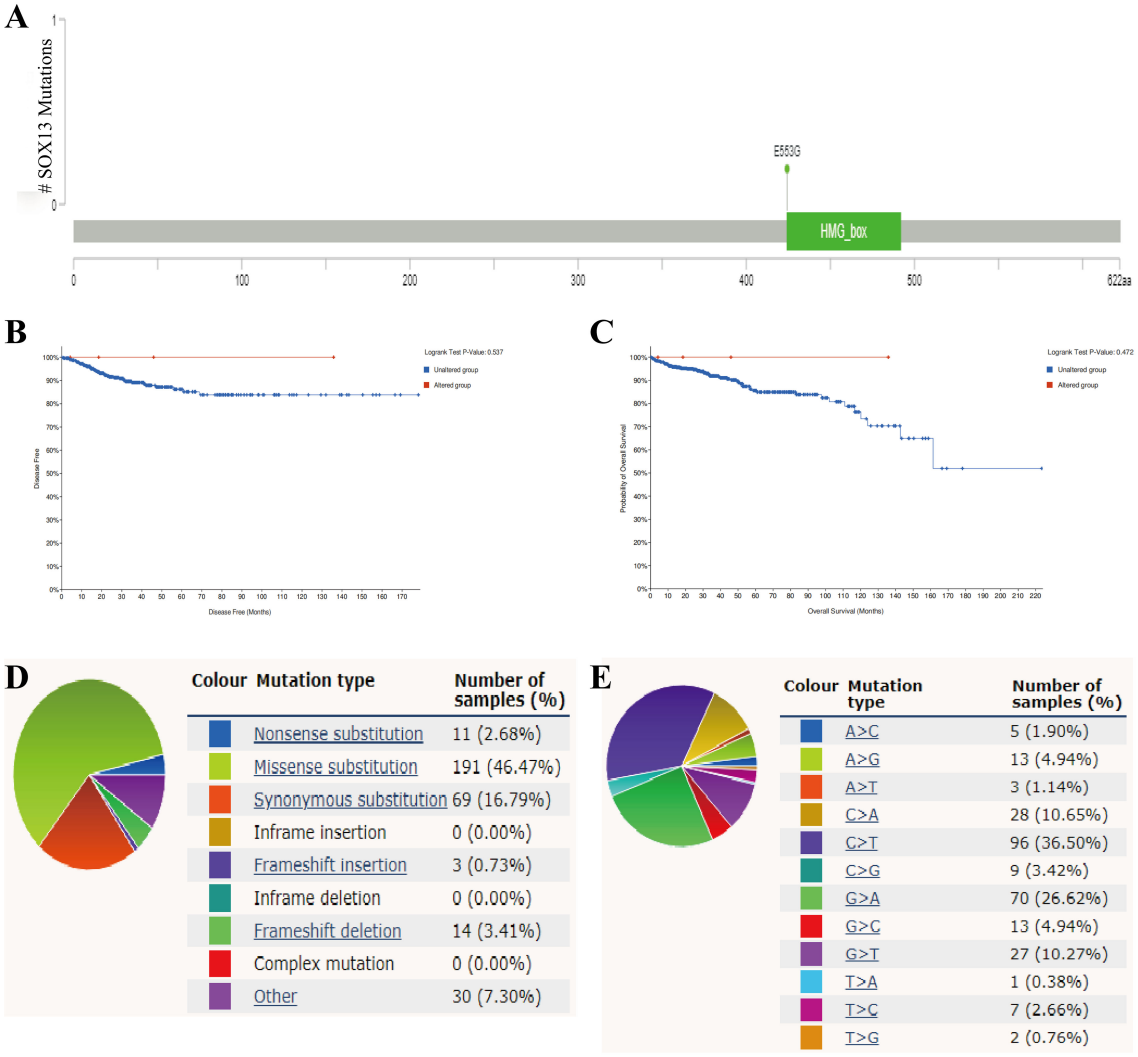
SOX13 mutations in THCA. **(A)** Schematic showing SOX13 mutations in THCA, determined by cBioPortal. **(B)** Potential association between SOX13 mutation status and disease-free survival of THCA, as evidenced by the cBioPortal tool. **(C)** Potential association between SOX13 mutation status and overall survival of THCA, as evidenced by the cBioPortal tool. **(D, E)** Types of SOX13 mutations, determined by the COSMIC.

Mutation frequencies in SOX13 were assessed using the cBioPortal online resource. All five datasets, namely, AMC, MSK, RIKEN, INSERM, and TCGA Pan-Cancer Atlas, were analyzed, comprising 1000 samples (21). The overall frequency of somatic mutations in SOX13 associated with THCA was found to be 0.2%, representing a low figure of just five mutations per 633 samples, most of which were missense mutations (**Figure 4A**). This indicated an absence of an association between SOX13 mutations and THCA patient prognosis (**Figures 4B, C**). The types of SOX13 mutations were also assessed using the COSMIC database. **Figure 4** shows two pie charts illustrating the different types of mutation. Approximately 46.47% of samples contained missense mutations, with synonymous mutations seen in 16.79% (**Figure 4D**). Most substitutions involved C > T (36.50%), with G > A accounting for 26.62% and C > A, 10.65% (**Figure 4E**).

### 3.4 Functional annotation and pathway enrichment of SOX13-associated genes within THCA

**Figure 5:**
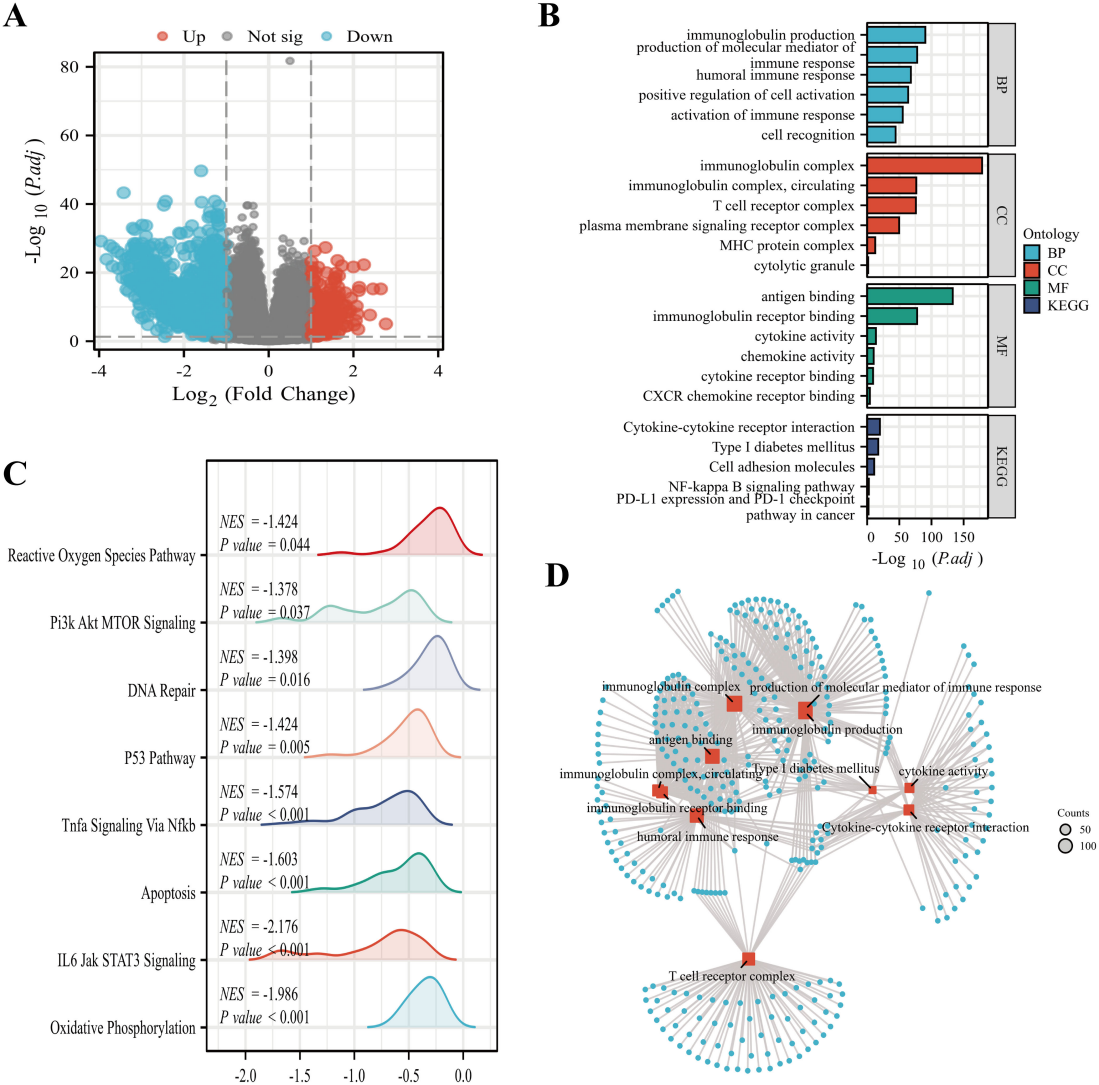
Functional enrichment analyses of SOX13-associated DEGs in THCA. **(A)** Volcano plot showing differential expression of SOX13^high^ and SOX13^low^ genes. **(B)** GO and KEGG analyses of SOX13-associated DEGs. **(C)** Analysis of the most significantly enriched networks between elevated SOX13 and reduced SOX13 samples using GSEA. **(D)** GO and KEGG analyses of SOX13-associated DEGs. *p<0.05; **p<0.01; ***p<0.001; ****p<0.0001. NS, not significant.

In all, we identified 1,659 DEGs using a threshold of |log2 fold-change (FC)| > 1.0 and adjusted p-value< 0.05, comprising 1,330 highly-expressed and 329 scarcely-expressed genes (**Figure 5A**). Subsequently, GO and KEGG analyses (**Figures 5B, D**) of DEGs were conducted, revealing primary biological processes (BP) including immunoglobulin synthesis, immune response mediator formation, and humoral immune response. The cellular component (CC) exhibited marked enrichment in the immunoglobulin complex, circulating immunoglobulin complex, and T cell receptor complex. The molecular function (MF) primarily involved antigen interaction, immunoglobulin receptor interaction, and cytokine activity. The enriched KEGG pathways were primarily related to cytokine-cytokine receptor association, Type I diabetes mellitus, and PD-L1 expression and PD-1 checkpoint. Finally, GSEA analysis was conducted on SOX13 differential genes and identified pathways, revealing enrichment in 8 pathways: reactive oxygen species, PI3K-AKT-mTOR, DNA repair, p53, oxidative phosphorylation, etc. (**Figure 5C**).

### 3.5 SOX13 is a major inverse modulator of ferroptosis in THCA

**Figure 6:**
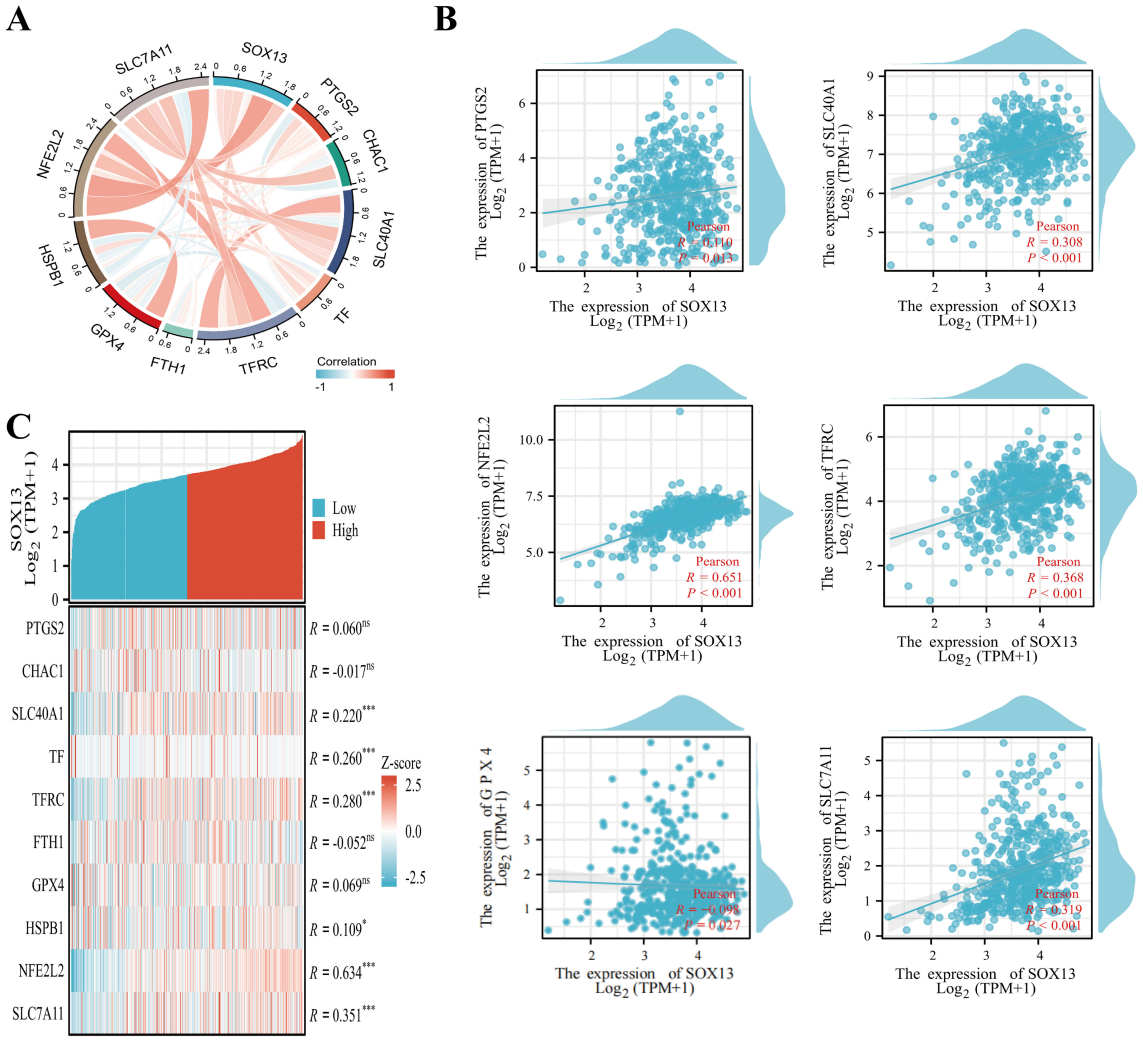
Relationships between the expression of SOX13 and that of ferroptosis-associated genes in THCA. **(A)** Correlations between SOX13 and ferroptosis markers using TCGA data. **(B)** Correlations between SOX13 and ferroptosis markers using GEPIA2 data. **(C)** Differentially expressed ferroptosis markers between the elevated- and reduced-SOX13 groups in THCA samples. *p<0.05; **p<0.01; ***p<0.001; ****p<0.0001. NS, not significant.

Prior investigations revealed that diminished oxidative phosphorylation and ROS contents suppress ferroptosis (22–24). Emerging reports reveal that ferroptosis suppression accelerates metastasis in clear cell renal cell carcinoma, breast cancer, and melanoma (25–27). Hence, we hypothesized that decreased SOX13 levels enables THCA metastasis via ferroptosis inhibition. To determine the association between SOX13 contents and ferroptosis, we conducted Pearson correlation analysis using GEPIA2 and TCGA-THCA datasets. This revealed a significant association between SOX13 levels and ferroptosis markers, such as SLC40A1, PTGS2, TFRC, GPX4, SLC7A11, and NFE2L2 (NRF2), with SOX13 showing the strongest link with NRF2 (R = 0.651, P< 0.001) (**Figures 6A, B**).

TCGA-THCA samples were stratified into reduced and elevated SOX13-expression groups, and differentially expressed ferroptosis-related genes were identified (**Figure 6C**). It was found that these genes, including SLC40A1, TF, TFRC, HSPB1, SLC7A11, and NFE2L2, were expressed strongly in the high-SOX13 group (P<0.05). Moreover, the cell viability of TPC-1 reduced markedly in correspondence with the RSL3 concentrations relative to the control (**Supplementary Figure S1A**). Overexpression of SOX13 enhances the inhibition of RSL3 (iron death activator) on the cell viability of TPC-1 (**Supplementary Figure S1B**). Thus, SOX13 may influence THCA progression and prognosis through its ability to regulate ferroptosis.

### 3.6 SOX13 levels and immune cell infiltration in THCA

**Figure 7:**
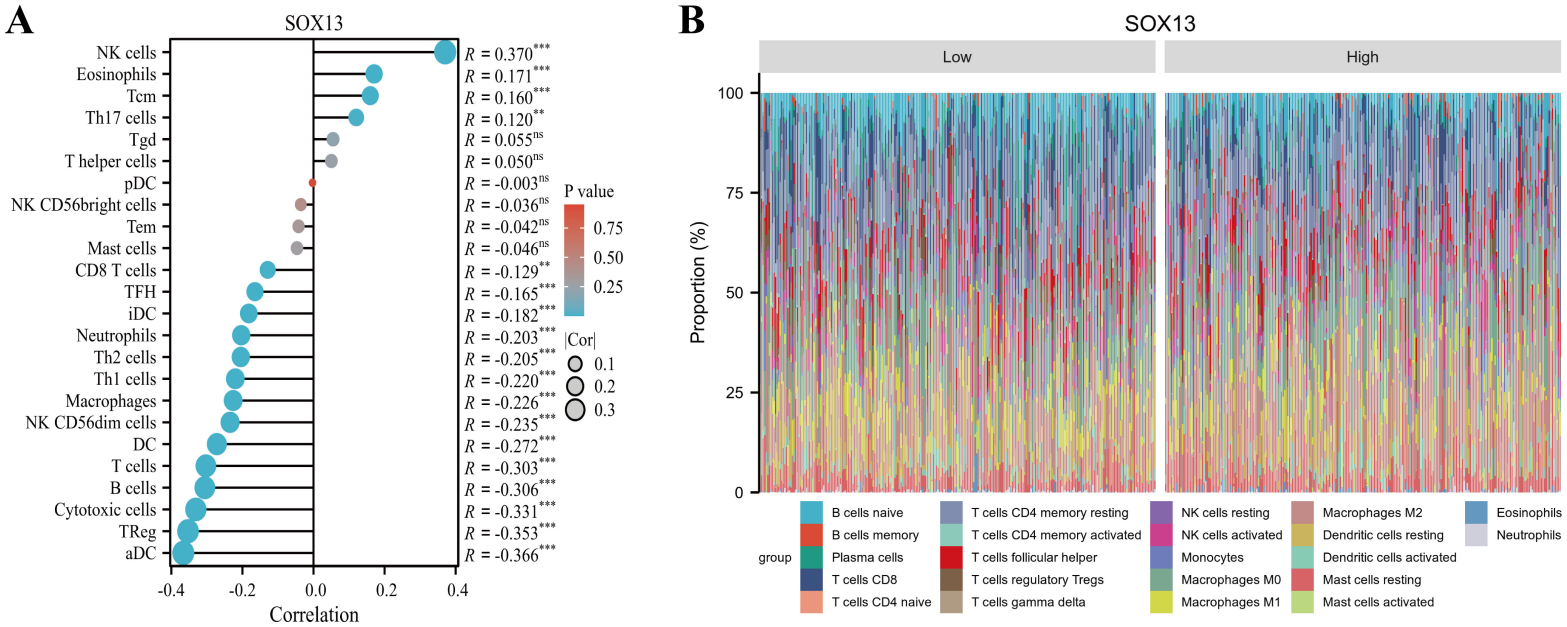
Relationships between SOX13 levels and immune cell invasion in THCA. **(A)** Correlations between 24 immune cell type invasions and SOX13 content using the ssGSEA algorithm. **(B)** Degree of 24 immune cell type invasions in elevated- and reduced-SOX13 cohorts assessed using the Cibersort algorithm.

Accumulating evidences indicate that cancer metabolism not only controls tumor development and progression but also reprograms immune cell metabolic networks, leading to dysregulated anti-tumor immune responses (28, 29). Currently, cancer metabolic regulation was shown to cooperatively augment immunotherapy in research (30). Therefore, herein, we investigated the association between SOX13 and tumor immunity, with the goal of determining its potential as an immunotherapy target. The tumor microenvironment (TME) is a critical modulator of tumor initiation and progression (31). In an immunosuppressive TME, dysfunctional infiltrating immune cells evade immune surveillance, leading to uncontrolled malignant cell proliferation (32–34). THCA tumor invasion by 24 immune cell types was assessed using ssGSEA, and their correlation with SOX13 levels was measured using Spearman’s correlation coefficients. We revealed that SOX13 content is directly related to multiple cell types, especially NK cells (R=0.370, P< 0.001), Eosinophils (R=0.171, P<0.001), Tcm (R=0.160, P<0.001), and type 17 T helper cells (R=0.120, P<0.01), while aDC (R=-0.366, P<0.001), regulatory T cell (R=-0.353, P<0.001), cytotoxic cells (R= -0331, P< 0.001), B cells (R=-0.306, P<0.001), and T cells (R= -0.303, P<0.001) were the strongest negatively related to SOX13 (**Figures 7A, B**).

**Figure 8:**
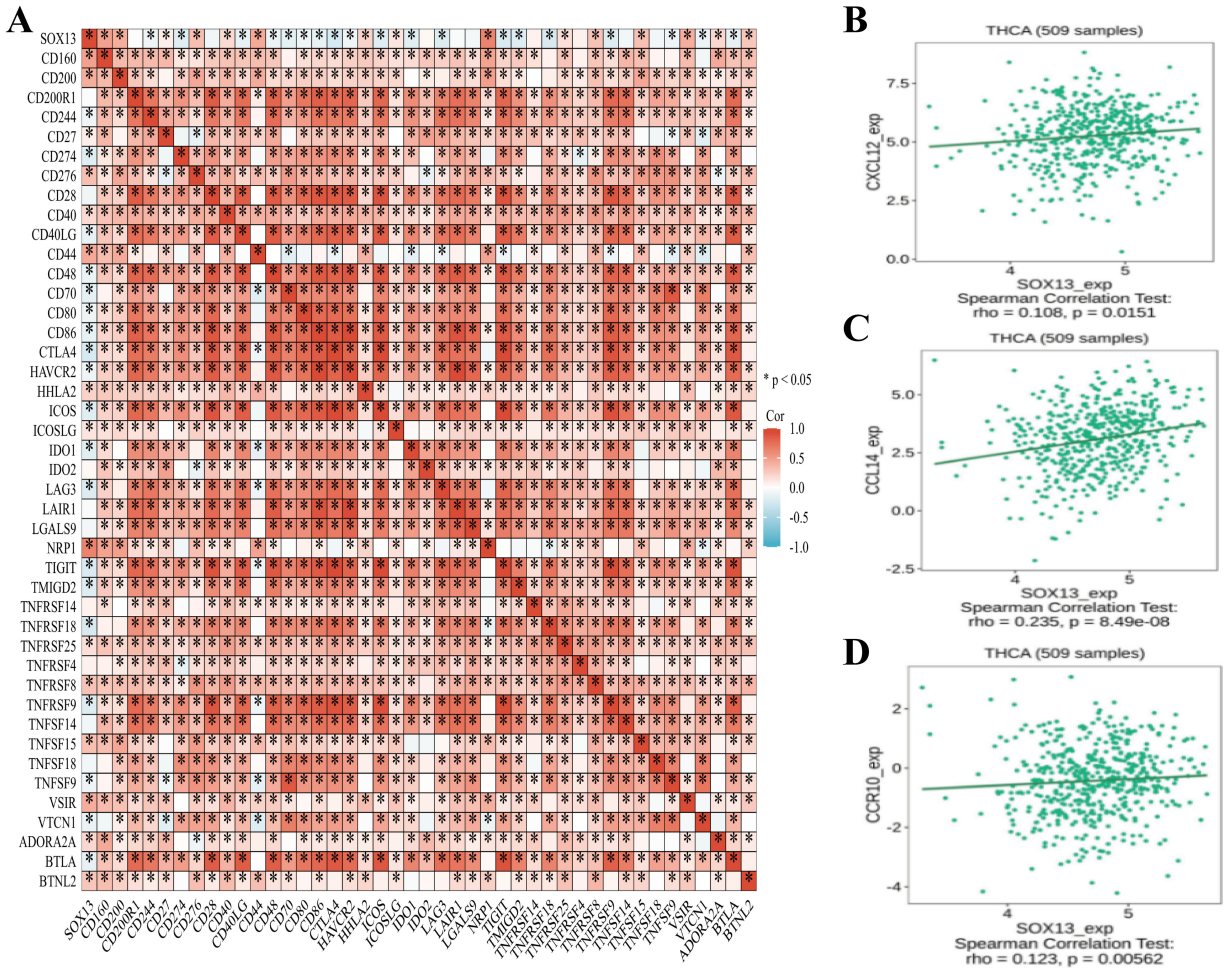
Associations between the SOX13, immune checkpoints, chemokines, and chemokine receptor contents. **(A)** Associations between levels of SOX13 and 40 immune checkpoints. **(B)** Direct relationships were seen between SOX13 and CXCL12 contents. **(C)** Direct relationships were seen between SOX13 levels and CCL14. **(D)** Positive correlations were seen between SOX13 and CCR10 contents.

Immunotherapy involving inhibition of immune checkpoints has shown promising results in many cancers (35). Thus, we evaluated associations between the levels of SOX13 and those of 40 genes encoding immune checkpoints genes in patients with THCA observing correlations between 36 of the 40 genes, such as CD200, CTLA4, CD276, and TNFRSF15 (**Figure 8A**). CTLA4 is known to be a biomarker associated with inhibition of immune checkpoints that could be used as a therapeutic target (36). These associations indicate that SOX13 may modulate the immune response in THCA. For further investigation into SOX13 and immune cell migrative property, this investigation subsequently probed relationships across SOX13 levels together with those of various chemokines and their receptors (**Figures 8B–D**). These results indicated that there were positive associations between SOX13 and CXCL12 contents (r = 0.108, P=0.0151), CCL14 (r = 0.235, P =8.49e−08), and CCR10 (r = 0.123, P=0.00562) in THCA. These findings thus suggest that SOX13 levels are linked with increased invasion of certain immune cell types within TME.

### 3.7 Upregulation of SOX13 suppresses THCA cell proliferation, migration, and invasion

**Figure 9:**
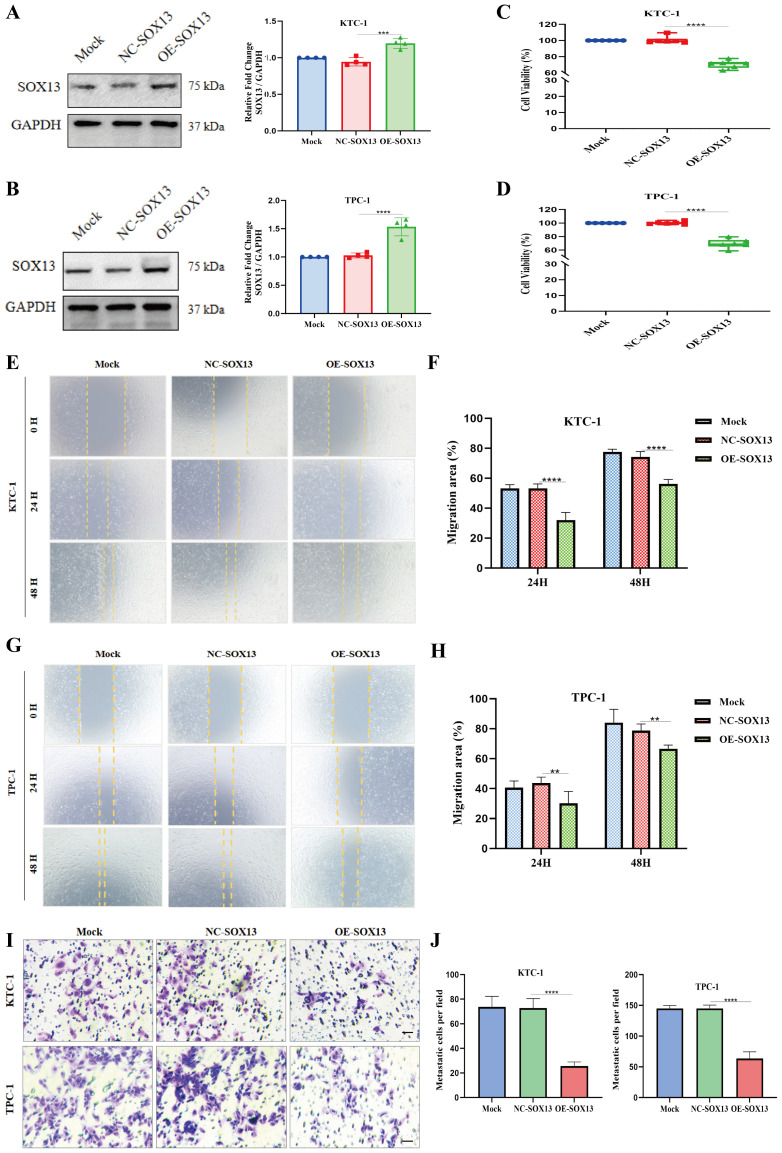
Upregulation of SOX13 reduces THCA cell proliferation, migration, and invasion. **(A, B)** SOX13 content significantly increased following SOX13 overexpression plasmid in two distinct THCA cell lines, as observed by Western blot (WB). Upregulation of SOX13 significantly inhibited proliferation **(C, D)**, migration **(E–H)**, and invasion **(I–J)** capabilities of THCA cells. Scale bar represents 50μm; **p< 0.01; ***p< 0.001;****p< 0.0001.

Western blotting confirmed a significant increase in SOX13 expression following transfection with the SOX13 overexpression plasmid in both KTC-1 and TPC-1 cells (**Figures 9A, B**). To further evaluate SOX13-mediated ferroptosis, the effects of ferroptosis-related genes were investigated by SOX13 overexpression plasmid in TPC-1 cells. Western blotting analysis revealed a significant up-regulation of SOX13 together with down-regulation of NRF2, GPX4, and SLC7A11 (**Supplementary Figure S2A–C**). Subsequently, CCK-8 tests demonstrated a remarkable decline in cell proliferation upon SOX13 overexpression (**Figures 9C, D**). Moreover, wound healing assays revealed a substantial decrease in metastatic potential in cells with activated SOX13 expression compared to the control group over the study duration (**Figures 9E–H**). Additionally, Transwell experiments were undertaken to assess the effects of SOX13 overexpression on cellular invasion capacity, demonstrating a significant reduction (**Figures 9I, J**).

## 4 Discussion

The SOX (SRY-related HMG-box) family was first discovered because of its highly conserved high-mobility motifs, and early studies focused on its role in embryonic development and tissue differentiation. However, the publication of numerous studies has led to an understanding of its role in tumorigenesis as well (37–39). SOX13 belongs to the D group within the SOX gene subfamily (40, 41). Within the SOXD subfamily, SOX13 was the most closely related to cancer development and progression. SOX13 critically regulates cell differentiation and stemness by modulating the Wnt/β-catenin axis (42). Emerging reports have indicated high SOX13 levels in liver cancer, with links to both metastasis and unfavorable prognosis (43). SOX13 is induced by hepatocyte growth factor through the JAK2/STAT3 signaling pathway, promoting metastasis in colorectal cancer by upregulating SNAI2 and c-MET expression (44). Additionally, research has shown high expression of SOX13 in various tumors including gastric (45), breast (46), and pancreatic cancers (47). Overall, these reports indicate that SOX13 has tumor-associated functions in multiple cancer types.

Nevertheless, there is limited information regarding SOX13 roles within THCA, whereby this investigation thus conducted a comprehensive bioinformatics-based investigation of its possible functional and diagnostic role in this context. This investigation revealed that SOX13 levels within tumor samples were markedly associated to tumor status and pathological phase, as well as survival outcomes. In addition, reduced SOX13 content was linked with worse patient prognosis and advanced tumor stages. SOX13 levels were shown by logistic regression as being markedly associated to T and pathologic stage, histological type, together with primary neoplasm focus type while Kaplan-Meier curves indicated that patient OS and PFI were markedly reduced in cases with lower SOX13 levels. Associations between SOX13 and poor prognosis have been observed in various cancer types, including liver cancer, gastric, nonsmall-cell lung cancer, and pancreatic cancers (48). Such dataset outcomes imply that SOX13 could be a prognostic biomarker within numerous cancers, including THCA.

The importance of genetic and epigenetic contributions to cancer development is well-known. For instance, mutations in the immune checkpoint gene PD-L1 influence its structure, cellular expression, and overall functioning (49). Overexpression of the JAK2/PDL1/PD-L2 pathway leads to activation and altered functioning of other immune checkpoint molecules (50, 51). However, here, it was found that SOX13 was mutated in only 0.2% of THCA tissue samples, nor were mutations linked with OS and DSS in THCA. Further investigations into possible SOX13 roles within THCA using annotation/pathway analyses indicated its involvement in various pathways including the activity of humoral immune response and the biology of cytokine-cytokine receptor interaction, suggesting that SOX13 may modulate immune response of tumor cells.

Targeting of ferroptosis has been suggested for treating cancer, especially for the treatment of refractory tumors (52). A number of tumor suppressor proteins, such as P53, fumarase, and BAP1, can sensitize tumor cells ferroptosis (53). Here, the relationships between SOX13 levels and ferroptosis-associated genes were investigated, observing positive correlations between them. These findings suggest that the SOX13-mediated tumorgenesis suppression is linked to the SOX13 ability to regulate ferroptosis and may reveal novel ferroptosis targeting in THCA treatment.

The TME encompasses not only tumor cells but also a diverse array of immune and stromal cells, namely fibroblasts, endothelial cells, and neurons (54). The immunogenicity of a tumor is influenced by its antigens, the extent of immune cell invasion, and the types and levels of immunomodulatory molecules present within the TME (55). Immune cell infiltration, a significant component of the TME, has been demonstrated to critically impact the initiation and advancement of cancers (56). Here, we identified an association between infiltration and SOX13 levels, observing that SOX13 was negatively associated with the numbers of cytotoxic, regulatory T cell, and B cells in THCA tumor samples. Cytotoxic cells initiate immune responses against tumors, resulting in tumor cell lysis (57), while regulatory T cells promote recruitment of type-1 T helper cells and immune activation (58). Our study also found a direct relationship between SOX13 content and emergence of type 17 helper T cells and Tcm cells. Type 17 helper T cells have been reported to suppress the glycolysis pathway, thereby intensifying inflammatory responses and affecting the progression of tumor diseases (59). Additionally, cytokines namely IL-4 and IL-13, secreted by Th2 cells, promote cancer progression by inducing polarization of M2 macrophages (60). SOX13 levels were also found to correlate with the concentrations of multiple chemokines, chemokine receptors, and immune checkpoints in THCA tissues, indicating its potential to influence the TME through diverse mechanisms. Our findings suggest that decreased levels of SOX13 are closely linked to mechanisms that facilitate immune evasion in THCA tumor cells, thereby contributing to both tumor growth and progression.

Finally, this study utilized SOX13 overexpression plasmid transfection to upregulate SOX13 expression in two THCA cell lines. The increased expression of SOX13 resulted in reduced viability, proliferation, metastasis, and invasion capabilities of THCA cells. Overexpression of SOX13 enhanced the inhibitory effect of RSL3 on the viability of TPC-1 cells. Moreover, our study also found that the overexpression of SOX13 inhibited the expression of ferroptosis-related genes in TPC-1 cells. This discovery further supports the involvement of SOX13 in the THCA cell cycle, regulating invasion and metastasis of THCA cells through ferroptosis. These findings highlight SOX13 as a promising target for targeted therapies against tumors.

This study provides crucial preliminary findings, but its sample population is relatively small. Future verification using a larger patient population is necessary. The accuracy of prediction and diagnosis using a single biomarker remains uncertain. Therefore, future research should prioritize combinations of multiple distinct biomarkers. Furthermore, the functional role of SOX13 in modulating immune response and ferroptosis was assessed using bioinformatics and *in vitro* cell experiments. To further validate these findings, *in vivo* tumor model experiments are necessary.

## 5 Conclusions

In conclusion, down-regulation of SOX13 may inhibit the antitumor immune response while promoting the incidence, metastasis, and invasion of THCA. SOX13 presence correlates with and has potential to predict the occurrence of THCA. These findings will assist medical professionals in developing more patient-friendly treatment regimens.

## Data Availability

The datasets presented in this study can be found in online repositories. The names of the repository/repositories and accession number(s) can be found in the article/**Supplementary Material**.
