# Supplementary material for: SOX13 as a potential prognostic biomarker linked to immune infiltration and ferroptosis inhibits the proliferation, migration, and metastasis of thyroid cancer cells
Source: Front Immunol. 2024 Dec 11;15:1478395. doi: 10.3389/fimmu.2024.1478395 (PMC11670200; doi:10.3389/fimmu.2024.1478395)
Supplement: Supplementary file 1 [file DataSheet1.docx]

**SOX13 as a Potential Prognostic Biomarker Linked to Immune Infiltration and Ferroptosis Inhibits the Proliferation, Migration, and Metastasis of Thyroid Cancer Cells**

Yan-yan Ma^1#^, Wei-ye Zhou^3#^, Yue Qian^4^, Ying-ying Mu^2*^, Wei Zhang^3,4*^

1. Department of Rehabilitation Medicine, Beijing Jishuitan Hospital Guizhou Hospital, Guiyang, 550000, Guizhou, China
2. Department of Pathology, Zunyi Hospital of Traditional Chinese Medicine, Zunyi, 563000, Guizhou, China
3. Cell Biology Department, Wuxi School of Medicine, Jiangnan University, Wuxi, 214122, Jiangsu, China
4. Department of Pathogen Biology, Guizhou Nursing Vocational College, Guiyang, 550000, Guizhou, China

#These authors contributed equally to this article

*Correspondence to:

Ying-ying Mu . Department of Pathology, Zunyi Hospital of Traditional Chinese Medicine, Zunyi, 563000, Guizhou, China. Email: zunyi_muyingying@163.com

Wei Zhang. Department of Pathogen Biology, Guizhou Nursing Vocational College, Guiyang, Guizhou, 550000, China. Email: [lawrence2013@163.com](mailto:lawrence2013@163.com)

**This file includes:** Supplementary Figures S1; S2

**Figure S1 Overexpression of SOX13 enhances the inhibition of RSL3 on the cell viability of TPC-1.**

1. Viability of TPC-1 cells after RSL3 exposure for 24H , assessed by CCK-8 assay. **(B)** Cell viability of TPC-1 cells treated with re-RSL3 for 24H after the transfected SOX13 overexpression plasmid 24H, by CCK-8 detection (n = 6, *p <0.05, **p < 0.01, ****p < 0.0001).

**Figure S2 Effect of overexpression of SOX13 on the expression of ferroptosis-related genes**

1. The expression of ferroptosis-related genes in TPC-1 cells treated for 48 h with the SOX13 overexpression plasmid. (B-C) Quantification of SOX13, NRF2, SLC7A11, TRFC, and GPX4 levels (n = 4, *p <0.05, ****p < 0.0001).

**Figure S1**

**
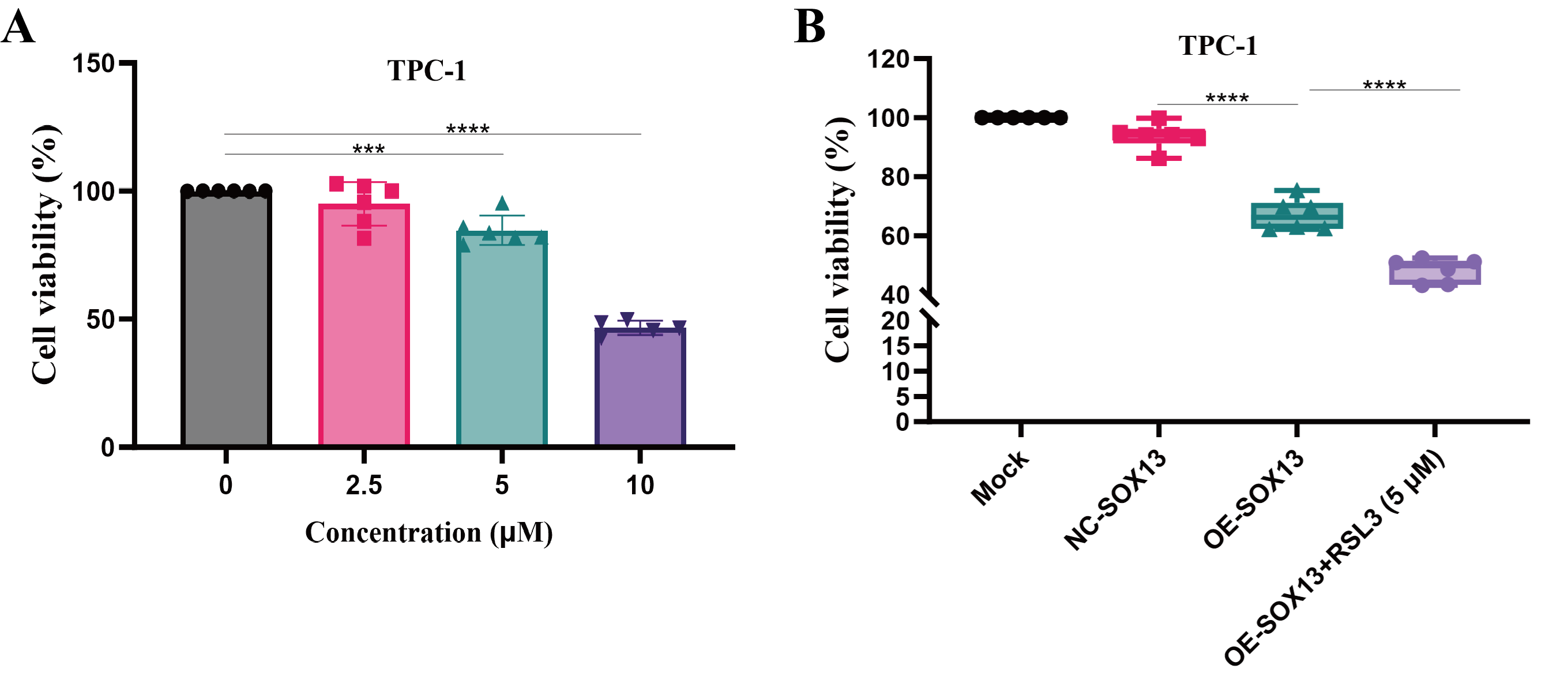
**

**Figure S2**

**
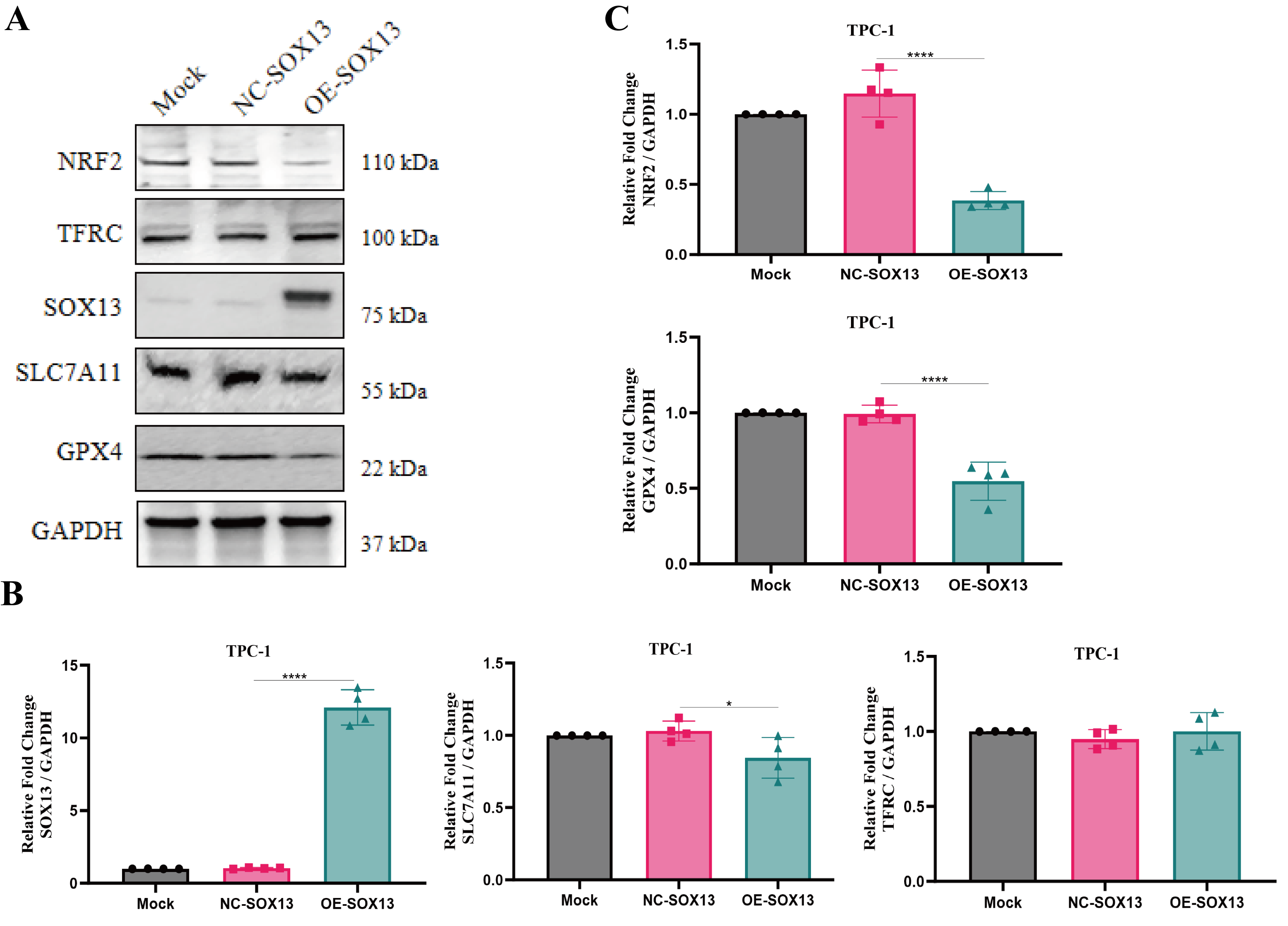
**
